# Two new species of *Pergalumna* (Acari, Oribatida, Galumnidae) from Costa Rica, including a key to all species of the genus from the Neotropical region

**DOI:** 10.3897/zookeys.435.8213

**Published:** 2014-08-15

**Authors:** Sergey G. Ermilov, Olman Alvarado-Rodríguez, Axel P. Retana-Salazar

**Affiliations:** 1 Tyumen State University, Tyumen, Russia; 2 Centro de Investigación en Estructuras, Microscópicas (CIEMIC), Ciudad de la Investigación, Universidad de Costa Rica, San José, Costa Rica

**Keywords:** Oribatid mites, new species, *Pergalumna*, key, Costa Rica, Neotropical region

## Abstract

Two new species of oribatid mites of the genus *Pergalumna* (Oribatida, Galumnidae), *P. elongatiporosa* **sp. n.** and *P. striatiprodorsum* **sp. n.**, are described from leaf litter of a secondary forest in Costa Rica. *Pergalumna elongatiporosa* **sp. n.** is most similar morphologically to *P. horvathorum* P. Balogh, 1997 and *P. sura* P. Balogh, 1997, however, it differs from both by the body size, body surface ornamentation and morphology of notogastral porose areas *A1* and *A3*. *Pergalumna striatiprodorsum* **sp. n.** is most similar morphologically to *P. hawaiiensis hawaiiensis* (Jacot, 1934) and *P. strigulata* Mahunka, 1978, however, it differs from *P. hawaiiensis* by the length of interlamellar setae and surface ornamentation of the prodorsum; from *P. strigulata* by the surface of ornamentation of the notogaster, length of interlamellar setae and morphology of bothridial setae. An identification key to known species of *Pergalumna* from the Neotropical region is given.

## Introduction

*Pergalumna* is a large genus that was proposed by Grandjean (1936) with *Oribata nervosa* Berlese, 1914 as type species. Currently, it comprises more than 130 species having a cosmopolitan distribution collectively (Subías 2004, updated 2014). The generic characters of the genus are summarized by Ermilov et al. (2013a), an identification key to some species has been presented by Balogh and Balogh (2002).

In the course of taxonomic identification of Costa Rican oribatid mites collected in 2013, we found two new species of the genus *Pergalumna*. The main goal of our paper is to describe these species. Earlier, only three species were known from Costa Rica (P. Balogh 1997; Schatz 2006, 2007; Ermilov et al. 2014): *Pergalumna horvathorum* P. Balogh, 1997, *Pergalumna silvatica* Hammer, 1961 and *Pergalumna sura* P. Balogh, 1997. An identification key to known species of *Pergalumna* from the Neotropical region is given in the present work.

## Materials and methods

Three specimens (holotype: male; two paratypes: one male and one female) of *Pergalumna elongatiporosa* sp. n. and nine specimens (holotype: female; eight paratypes: five males and three females) of *Pergalumna striatiprodorsum* sp. n. are from: Costa Rica, 9°50'24"N, 83°53'17"W, Cartago, Dulce Nombre, Paraíso, Jardín Botánico Lankester, 1400 m a.s.l., in leaf litter in secondary forest, 14.V.2013, collected by O. Alvarado-Rodríguez and A.P. Retana-Salazar.

Holotypes and paratypes were mounted in lactic acid on temporary cavity slides for measurement and illustration. The body length was measured in lateral view, from the tip of the rostrum to the posterior edge of the ventral fig. The notogastral width refers to the maximum width in dorsal aspect. Lengths of body setae were measured in lateral aspect. All body measurements are presented in micrometers. Formulae for leg setation are given in parentheses according to the sequence trochanter–femur–genu–tibia–tarsus (famulus included). Formulae for leg solenidia are given in square brackets according to the sequence genu–tibia–tarsus. General terminology used in this paper follows that of Grandjean (summarized by Norton and Behan-Pelletier 2009).

## Taxonomy

### Pergalumna elongatiporosa sp. n.

Taxon classificationAnimaliaOribatidaGalumnidae

http://zoobank.org/E008B3A2-4238-4C4E-B918-8B9E10E48686

Figs 1 – 4

#### Diagnosis.

Body size: 332–352 × 246–266. Body surface and pteromorphs microgranulate. Rostral, lamellar and interlamellar setae well developed, barbed. Bothridial setae setiform, ciliate unilaterally. Anterior notogastral margin not developed. Three pairs of porose areas: *Aa* and *A3* elongate triangular, *A1* long, band-shaped, specifically curving, *Aa* located between notogastral alveoli *la* and *lm*. Median pore and postanal porose area absent. Aggenital and ano-adanal setae simple, short. Solenidion φ on tibia IV inserted in proximal part.

#### Description.

*Measurements*. Body length: 332 (holotype, male), 340–352 (two paratypes: one male and one female); notogaster width: 246 (holotype), 246–266 (two paratypes).

*Integument*. Body color brown to black-brown. Body surface and pteromorphs with dense microgranules (their diameter up to 2). Pteromorphs with poorly visible wrinkles.

*Prodorsum*. Rostrum broadly rounded. Rostral (*ro*, 24–28), lamellar (*le*, 49–53) and interlamellar (*in*, 69–77) setae setiform, barbed. Bothridial setae (*ss*, 90–102) setiform, densely ciliate unilaterally. Exobothridial setae absent. Lamellar and sublamellar lines distinct, parallel, curving backwards. Insertions of lamellar setae distanced from the lamellar lines. Porose areas *Ad* small, elongate oval (8–12 × 2–4), located latero-posteriorly to interlamellar setae.

*Notogaster*. Anterior notogastral margin not developed. Dorsophragmata (*D*) long. Notogastral setae represented by 10 pairs of alveoli. Three pairs of porose areas well visible, with distinct margins: *Aa* weakly triangular, transversally oriented (36–57 × 8–12), *A1* long, band-shaped, specifically curving (57–69 × 8–13), *A3* elongate, narrowly triangular (28–32 × 8–16). Porose areas *Aa* located between notogastral alveoli *la* and *lm*. Median pore absent. All lyrifissures distinct; *im* and opisthonotal gland openings (*gla*) located latero-anteriorly to *A1*.

*Gnathosoma*. Morphology of subcapitulum, palps and chelicerae typical for *Pergalumna* (see Engelbrecht 1972; Ermilov et al. 2010; Ermilov and Anichkin 2011). Subcapitulum longer than wide (90–94 × 77–86). Subcapitular setae setiform, slightly barbed; *a* (14–16) longer than *m* (10) and *h* (8). Two pairs of adoral setae (*or*_1_, *or*_2_, 8) setiform, hook-like distally, barbed. Palps (73) with setation 0–2–1–3–9(+ω). Solenidion attached to eupathidium, both located on dorsal tubercle. Chelicerae (118–123) with two setiform, barbed setae; *cha* (28–32) longer than *chb* (20). Trägårdh’s organ distinct.

*Epimeral and lateral podosomal regions*. Apodemes 1, 2, sejugal and 3 well visible. Six pairs of setiform epimeral setae observed; setal formula: 1–0–2–3. Setae *4a*, *4b* (4) thin, smooth, shorter than *1b*, *3b*, *3c*, *4c* (8–10), slightly barbed. Pedotecta II (Pd II) scale-like, rounded in ventral view. Discidia (*dis*) pointly triangular. Circumpedal carinae (*cp*) distinct, directed posterior of setae *3b*.

*Anogenital region*. Six pairs of genital (*g*_1_, *g*_2_, 8; *g*_3_–*g*_6_, 4), one pair of aggenital (*ag*, 4), two pairs of anal (*an*_1_, *an*_2_, 4) and three pairs of adanal (*ad*_1_–*ad*_3_, 4) setae minute, thin, smooth. Anterior parts of genital figs with two setae. Adanal setae *ad*_3_ inserted laterally or antero-laterally to lyrifissures *iad*. Postanal porose area absent.

*Legs*. Morphology of leg segments, setae and solenidia typical for *Pergalumna* (see Engelbrecht 1972; Ermilov et al. 2010; Ermilov and Anichkin 2011). Formulae of leg setation and solenidia: I (1–4–3–4–20) [1–2–2], II (1–4–3–4–15) [1–1–2], III (1–2–1–3–15) [1–1–0], IV (1–2–2–3–12) [0–1–0]; homology of setae and solenidia indicated in Table 1. Solenidion φ on tibia IV inserted in proximal part.

**Figure 1.**
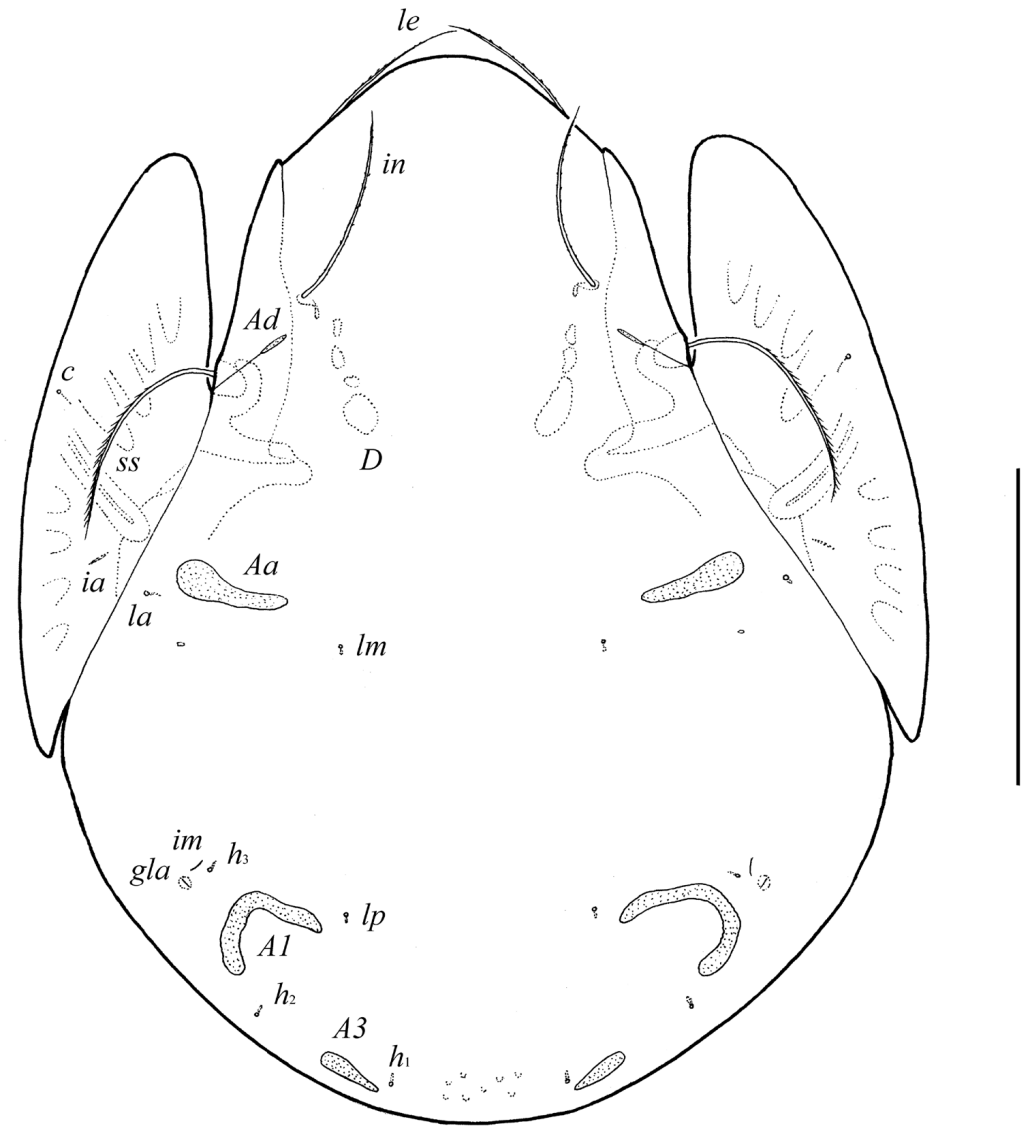
*Pergalumna elongatiporosa* sp. n.: dorsal view. Scale bar 100 μm.

**Figure 2.**
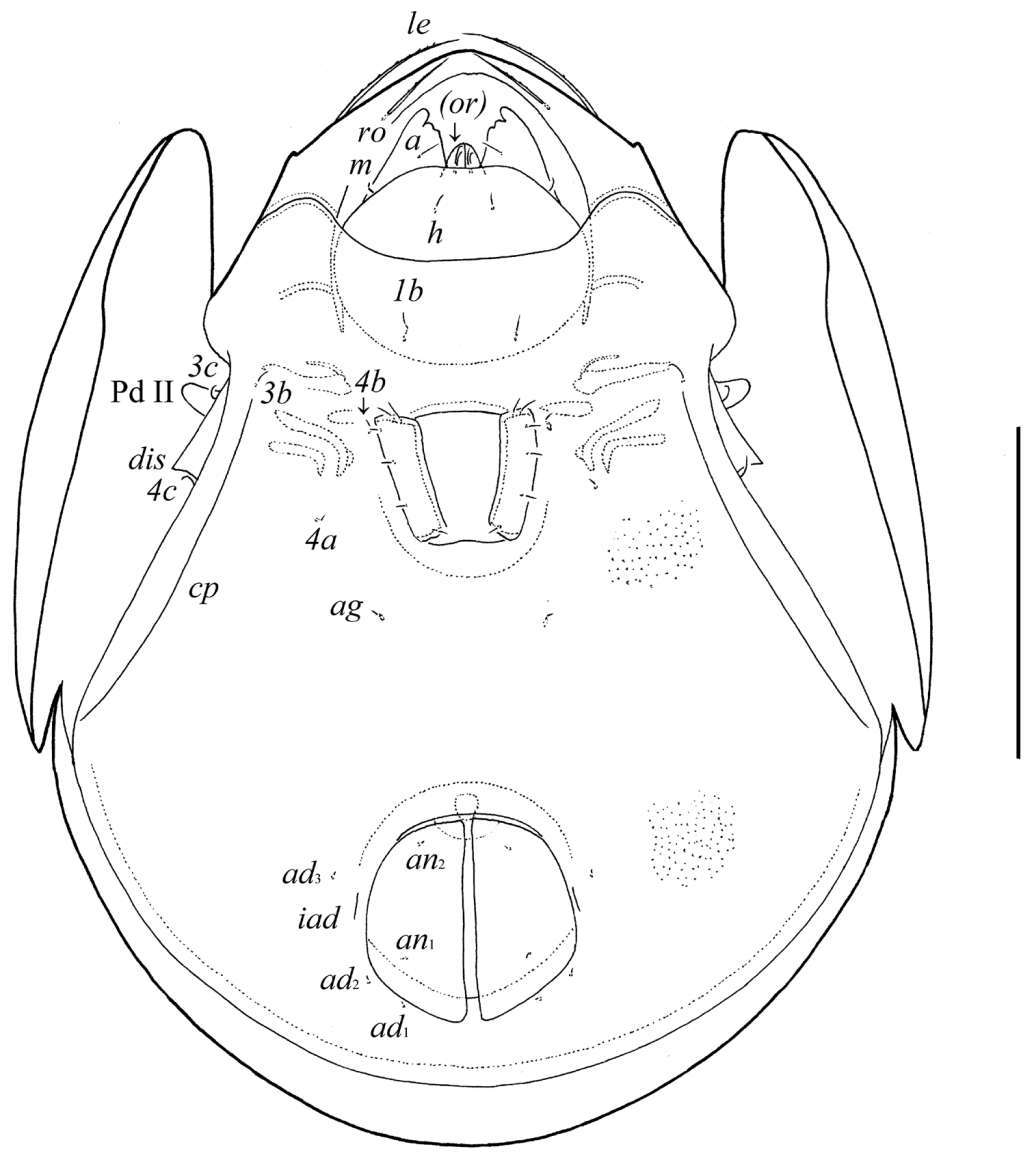
*Pergalumna elongatiporosa* sp. n.: ventral view (legs not illustrated). Scale bar 100 μm.

**Figures 3–4.**
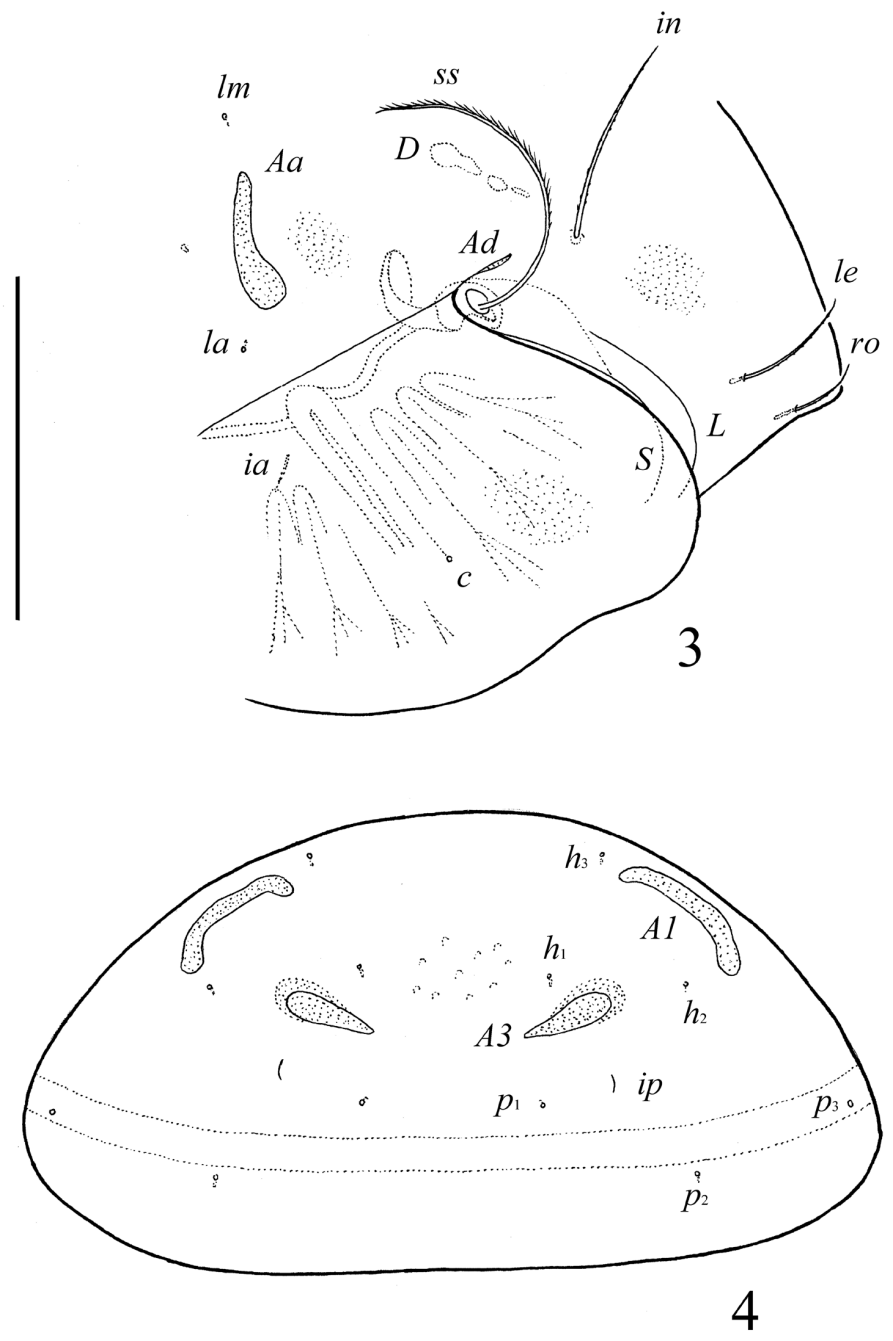
*Pergalumna elongatiporosa* sp. n.: **3** dorso-lateral view of prodorsum and anterior part of notogaster and pteromorph (gnathosoma and legs not illustrated) **4** posterior view of notogaster. Scale bars 100 μm.

**Table 1.** Leg setation and solenidia of *Pergalumna elongatiporosa* sp. n. (same data for *Pergalumna striatiprodorsum* sp. n.).

Leg	Trochanter	Femur	Genu	Tibia	Tarsus
I	*v*’	*d*, *(l)*, *bv*’’	*(l)*, *v*’, σ	*(l)*, *(v)*, φ_1_, φ_2_	*(ft)*, *(tc)*, *(it)*, *(p)*, *(u)*, *(a)*, *s*, *(pv)*, *v’*, *(pl)*, *l*’’, ε, ω_1_, ω_2_
II	*v*’	*d*, *(l)*, *bv*’’	*(l)*, *v*’, σ	*(l)*, *(v)*, φ	*(ft)*, *(tc)*, *(it)*, *(p)*, *(u)*, *(a)*, *s*, *(pv)*, ω_1_, ω_2_
III	*v*’	*d*, *ev*’	*l*’, σ	*l’*, *(v)*, φ	*(ft)*, *(tc)*, *(it)*, *(p)*, *(u)*, *(a)*, *s*, *(pv)*
IV	*v*’	*d*, *ev*’	*d*, *l*’	*l’*, *(v)*, φ	*ft’’*, *(tc)*, *(p)*, *(u)*, *(a)*, *s*, *(pv)*

Roman letters refer to normal setae (ε to famulus), Greek letters to solenidia. Single prime (’) marks setae on anterior and double prime (’’) setae on posterior side of the given leg segment. Parentheses refer to a pair of setae.

#### Type deposition.

The holotype is deposited in the Zoological Institute of the Russian Academy of Sciences, St. Petersburg, Russia; one paratype is deposited in the collection of the Tyumen State University Museum of Zoology, Tyumen, Russia; one paratype is deposited in the collection of the Centro de Investigación en Estructuras, Microscópicas (CIEMIC), Ciudad de la Investigación, Universidad de Costa Rica, San José, Costa Rica.

#### Etymology.

This specific name “*elongatiporosa*” refers to the elongate porose areas *A1*.

#### Remarks.

In having the setiform bothridial setae, well developed interlamellar setae, absence of anterior notogastral margin and presence of three pairs of porose areas (*Aa* transversally elongated), *Pergalumna elongatiporosa* sp. n. is most similar to *Pergalumna horvathorum* P. Balogh, 1997 and *Pergalumna sura* P. Balogh, 1997 (see also Ermilov et al. 2014) from the Neotropical region. However, it differs from both by the smaller body size (332–352 × 246–266 versus 394–410 × 295 in *Pergalumna horvathorum* and 443–498 × 315–377 in *Pergalumna sura*), microgranulate body surface (versus smooth in *Pergalumna horvathorum* and *Pergalumna sura*), band-shaped, specifically curving porose areas *A1* (versus elongate oval in *Pergalumna horvathorum* and triangular in *Pergalumna sura*) and elongate triangular porose areas *A3* (versus absent in *Pergalumna horvathorum* and oval in *Pergalumna sura*).

### Pergalumna striatiprodorsum sp. n.

Taxon classificationAnimaliaOribatidaGalumnidae

http://zoobank.org/2DBFC49F-0502-4A55-AC59-6E1E45C8358C

Figs 5 – 8

#### Diagnosis.

Body size: 630–697 × 448–514. Body surface and pteromorphs microgranulate; surface of prodorsum with numerous longitudinal stria. Rostral, lamellar and bothridial setae of medium size, setiform, barbed; interlamellar setae short, slightly thickened, barbed. Anterior notogastral margin not developed. Three pairs of porose areas oval; *Aa* located between notogastral alveoli *la* and *lm*, close to *lm*. Median pore absent. Aggenital and ano-adanal setae simple, short. Postanal porose area present, elongated.

#### Description.

*Measurements*. Body length: 697 (holotype, female), 630–697 (eight paratypes: five males and three females); notogaster width: 514 (holotype), 448–514 (eight paratypes).

*Integument*. Body color brown to black-brown. Body surface, pteromorphs and subcapitular mentum with dense microgranules (their diameter up to 2). Surface of prodorsum with numerous longitudinal stria. Pteromorphs with poorly visible wrinkles.

*Prodorsum*. Rostrum broadly rounded. Rostral (41–53), lamellar (57–69) and bothridial (106–114) setae setiform, barbed. Interlamellar setae short (12–16), setiform, slightly thickened, barbed. Exobothridial setae absent. Lamellar and sublamellar lines distinct, parallel, curving backwards. Insertions of lamellar setae distanced from the lamellar lines. Porose areas *Ad* elongate oval (20–28 × 4–8), located latero-posteriorly to interlamellar setae.

*Notogaster*. Anterior notogastral margin not developed. Dorsophragmata of medium size. Notogastral setae represented by 10 pairs of alveoli. Three pairs of porose areas well visible, with distinct margins: *Aa* rounded (14–16) or oval, weakly transversally oriented (14–20 × 12–16), *A1* oval, weakly elongated diagonally (24–41 × 12–20), *A3* rounded (12–16 )or oval (12–16 × 10–12). Porose areas *Aa* located between notogastral alveoli *la* and *lm*, but clearly closer to *lm*. Median pore absent. All lyrifissures distinct; *im* located latero-anteriorly to *A1*. Opisthonotal gland openings located laterally to *A1*.

*Gnathosoma*. Morphology of subcapitulum, palps and chelicerae typical for *Pergalumna* (see Engelbrecht 1972; Ermilov et al. 2010; Ermilov and Anichkin 2011). Subcapitulum longer than wide (155–159 × 131–143). Subcapitular setae setiform, slightly barbed; *a* (24) longer than *m* (16) and *h* (14–16). Two pairs of adoral setae (12–14) setiform, hook-like distally, barbed. Palps (123–127) with setation 0–2–1–3–9(+ω). Solenidion attached to eupathidium, both located on dorsal tubercle. Chelicerae (196–205) with two setiform, barbed setae; *cha* (53) longer than *chb* (32–36). Trägårdh’s organ distinct.

*Epimeral and lateral podosomal regions*. Apodemes 1, 2, sejugal and 3 well visible. Six pairs of setiform epimeral setae observed; setal formula: 1–0–2–3. Setae *4a*, *4b* (6–8) thin, smooth, shorter than *3b* (20–24) and *1b*, *3c*, *4c* (32–36), slightly barbed. Pedotecta II scale-like, rounded in ventral view. Discidia pointly triangular. Circumpedal carinae distinct, directed posterior of setae *3b*.

*Anogenital region*. Six pairs of genital (*g*_1_, 16–18; *g*_2_, 12–14; *g*_3_–*g*_6_, 8–10), one pair of aggenital (6–8), two pairs of anal (6–8) and three pairs of adanal (6–8) setae setiform, thin, smooth. Anterior parts of genital figs with two setae. Adanal setae *ad*_3_ inserted laterally to lyrifissures *iad*. Postanal porose area present, elongate oval (20–28 × 6–8).

*Legs*. Morphology of leg segments, setae and solenidia typical for *Pergalumna* (see Engelbrecht 1972; Ermilov et al. 2010; Ermilov and Anichkin 2011). Formulae of leg setation and solenidia: I (1–4–3–4–20) [1–2–2], II (1–4–3–4–15) [1–1–2], III (1–2–1–3–15) [1–1–0], IV (1–2–2–3–12) [0–1–0]; homology of setae and solenidia indicated in Table 1.

**Figure 5.**
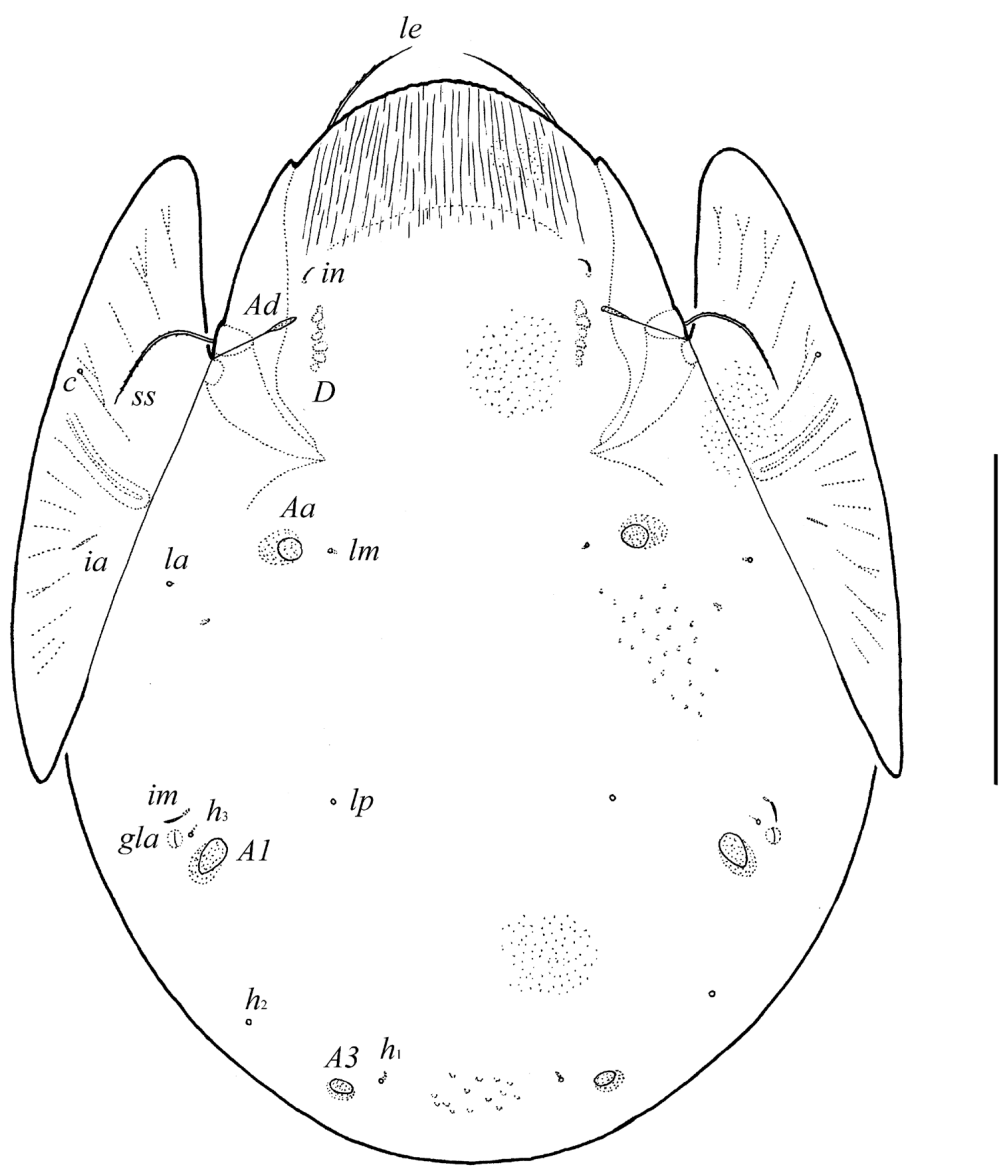
*Pergalumna striatiprodorsum* sp. n.: dorsal view. Scale bar 200 μm.

**Figure 6.**
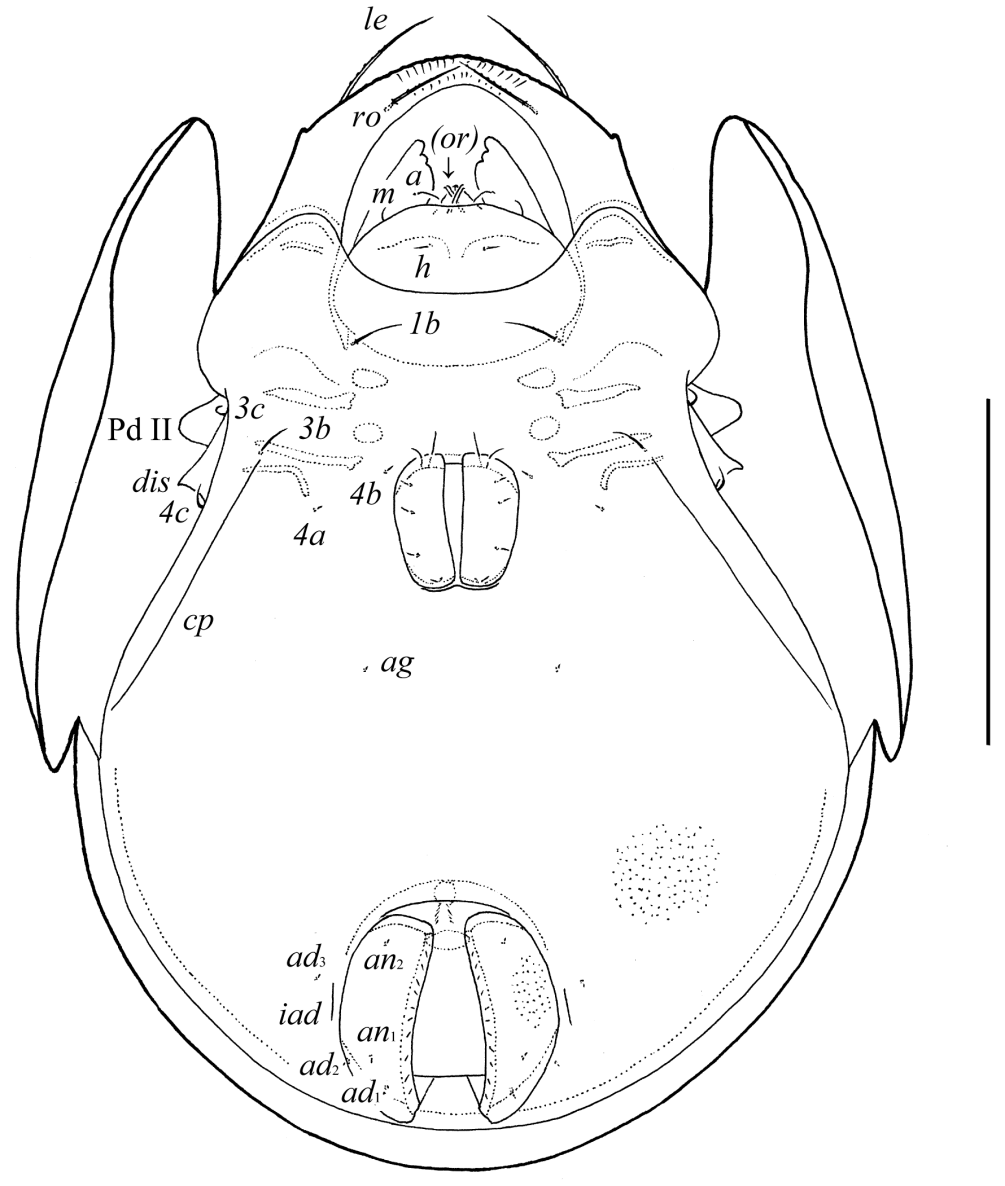
*Pergalumna striatiprodorsum* sp. n.: ventral view (legs not illustrated). Scale bar 200 μm.

**Figures 7–8.**
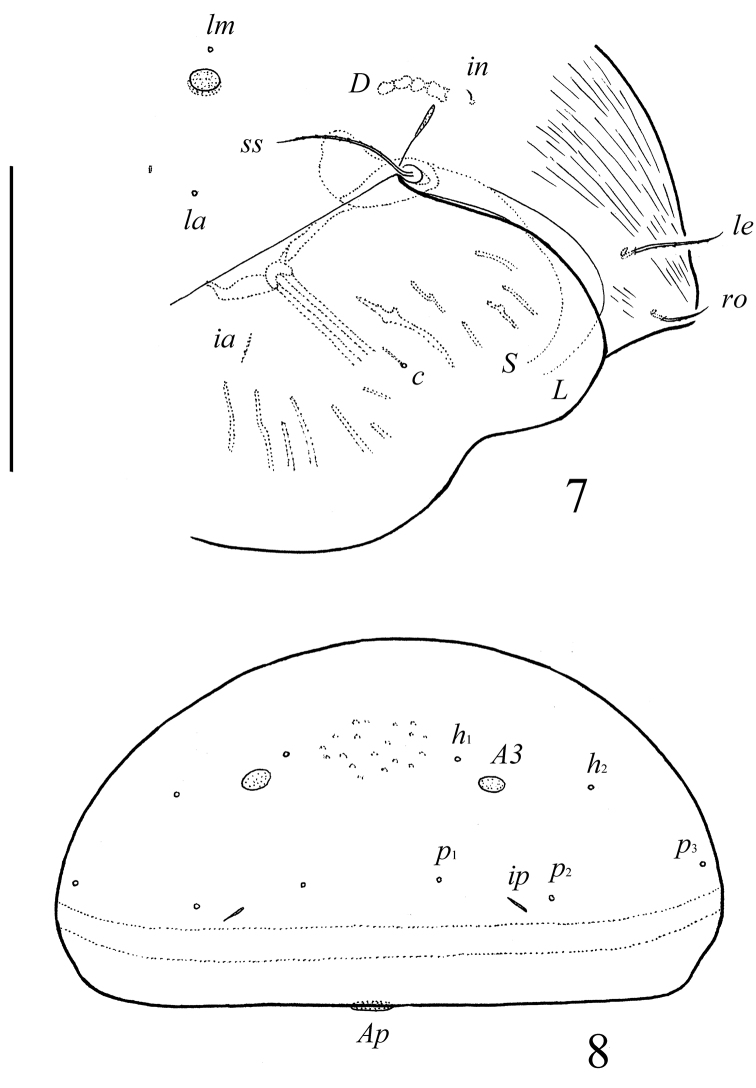
*Pergalumna striatiprodorsum* sp. n.: **7** dorso-lateral view of prodorsum and anterior part of notogaster and pteromorph (gnathosoma and legs not illustrated) **8** posterior view of notogaster. Scale bars 200 μm.

#### Type deposition.

The holotype is deposited in the Zoological Institute of the Russian Academy of Sciences, St. Petersburg, Russia; seven paratypes are deposited in the collection of the Tyumen State University Museum of Zoology, Tyumen, Russia; one paratype is deposited in the collection of the Centro de Investigación en Estructuras, Microscópicas (CIEMIC), Ciudad de la Investigación, Universidad de Costa Rica, San José, Costa Rica.

#### Etymology.

This specific name “*striatiprodorsum*” refers to the striate prodorsum.

#### Remarks.

In having the striate prodorsum, setiform bothridial setae, absence of anterior notogastral margin and presence of three pairs of oval porose areas (*Aa* oval, rounded, located close to *lm*), *Pergalumna striatiprodorsum* sp. n. is most similar to *Pergalumna hawaiiensis hawaiiensis* (Jacot, 1934) from the Pacific Islands and *Pergalumna strigulata* Mahunka, 1978 from Mauritius. However, it differs from *Pergalumna hawaiiensis* by the short interlamellar setae (versus medium size in *Pergalumna hawaiiensis*) and striate prodorsum (versus only anterior part of prodorsum with stria in *Pergalumna hawaiiensis*); from *Pergalumna strigulata* by the absence of stria on notogaster (versus notogaster striate in *Pergalumna strigulata*), short interlamellar setae (versus medium size in *Pergalumna strigulata*) and barbed bothridial setae (versus smooth in *Pergalumna strigulata*).

Among Neotropical species *Pergalumna striatiprodorsum* sp. n. is most similar morphologically to *Pergalumna decorata* Balogh & Mahunka, 1977. However, it differs from the latter by the absence of anterior notogastral margin (versus anterior margin present in *Pergalumna decorata*), barbed bothridial setae (versus smooth in *Pergalumna decorata*), absence of stria on notogaster (versus notogaster striate in *Pergalumna decorata*) and microgranulate body surface and pteromorphs (versus with larger foveoles in *Pergalumna decorata*).

### Key to known species of *Pergalumna* from the Neotropical region^1^

**Table:** 

1	Bothridial setae with clear head	2
–	Bothridial setae setiform	13
2	Anterior margin of notogaster developed	3
–	Anterior margin of notogaster not developed	9
3	Surface of prodorsum with numerous longitudinal stria or rugae	4
–	Surface of prodorsum without stria and rugae	5
4	Surface of notogaster striate; interlamellar setae of medium size; notogaster with four pairs of porose areas (*A2* present); body length: 330–460	*Pergalumna striata* (Pérez-Íñigo & Baggio, 1980) (see also Pérez-Íñigo and Baggio 1994). Distribution: Brazil
–	Surface of notogaster foveolate; interlamellar setae absent; notogaster with three pairs of porose areas (*A2* absent); body size: 486–527 × 405–437	*Pergalumna complicata* Balogh & Mahunka, 1978 (see Fig. 23A–B, not Fig. 24A–E, in Balogh and Mahunka 1978). Distribution: Brazil
5	Centro-anterior part of notogaster with specific ornamentation (longitudinal line with lateral lineate branches); lamellar setae minute; notogastral porose areas *Aa* elongated transversally, very narrow; body size: 282–298 × 199–215	*Pergalumna ornamenta* Ermilov, Starý, Sandmann, Marian & Maraun, 2013. Distribution: Ecuador
–	Centro-anterior part of notogaster without specific ornamentation; lamellar setae of medium size or long; notogastral porose areas *Aa* triangular, boot-shaped, rounded or oval	6
6	Interlamellar setae long; notogastral porose areas *Aa* triangular or boot-shaped	7
–	Interlamellar setae minute; notogastral porose areas *Aa* rounded or oval	8
7	Median pore located posterior to the virtual line connecting porose areas *A2*; notogastral porose areas *A1* and *A2* small; lyrifissures *im* located between setal alveoli *lm* and *lp*; body length: 470–600	*Pergalumna bryani bryani* (Jacot, 1934) (see also Hammer 1973). Distribution: Pacific Islands and Galapagos Islands
–	Median pore located little posterior to the virtual line connecting porose areas *A1*; notogastral porose areas *A1* and *A2* of medium size; lyrifissures *im* located laterally to *A1*; body size: 620 × 430	*Pergalumna comparanda* (Berlese, 1920) (see also Mahunka 1992). Distribution: Argentina
8	Bothridial setae pointed distally; notogastral porose areas *A3* elongated, longer than *A1* and *A2*; body length: 820	*Pergalumna andicola* Hammer, 1961. Distribution: Peru
–	Bothridial setae rounded distally; notogastral porose areas *A1*, *A2* and *A3* rounded, similar in size; body length: 730	*Pergalumna anellata* Hammer, 1961. Distribution: Peru
9	Interlamellar setae long	10
–	Interlamellar setae minute or represented by alveoli	11
10	Bothridial head barbed medio-distally, stalk smooth; rostral setae inserted laterally; pteromorphal wrinkles indistinct; body size: 320–400 × 270	*Pergalumna numerosa* (Sellnick, 1923) (see also see also Pérez-Íñigo and Baggio 1994). Distribution: Brazil
–	Bothridial head and stalk barbed; rostral setae inserted ventro-laterally; pteromorphal wrinkles clearly visible; body size: 338–392 × 258–320	*Pergalumna bellesii* Pérez-Íñigo & Baggio, 1997. Distribution: Brazil
11	Basal part of prodorsum with longitudinal stria; notogaster with four pairs of porose areas; lamellar setae of medium size; body length: 600	*Pergalumna montana* Hammer, 1961. Distribution: Peru and Venezuela
–	Basal part of prodorsum without longitudinal stria; notogaster with three pairs of porose areas; lamellar setae short	12
12	Notogastral porose areas *Aa* oval; median pore present; bothridial setae well barbed; body length: 492–576	*Pergalumna nasica* Pérez-Íñigo & Baggio, 1980. Distribution: Brazil and Argentina
–	Notogastral porose areas *Aa* triangular; bothridial setae indistinctly barbed; median pore absent; body length: 468 × 360	*Pergalumna cardosensis* Pérez-Íñigo & Baggio, 1986. Distribution: Brazil and Peru
13	Anterior margin of notogaster developed	14
–	Anterior margin of notogaster not developed	17
14	Surface of prodorsum and notogaster nearly smooth; body size: 520–676 × 502	*Pergalumna foveolata* Hammer, 1973 (see also Bayartogtokh and Chattarjee 2010). Distribution: Polynesia, India and Brazil
–	Surface of prodorsum with longitudinal stria or large foveoles; surface of notogaster striate	15
15	Surface of prodorsum with longitudinal stria; interlamellar setae minute; notogastral porose areas *Aa* located closer to setal alveoli *lm* than to *la*; body size: 637–653 × 469–494	*Pergalumna decorata* Balogh & Mahunka, 1977. Distribution: Neotropical region
–	Surface of prodorsum with large foveoles; interlamellar setae of medium size; notogastral porose areas *Aa* equal distanced from setal alveoli *la* and *lm*	16
16	Rostrum pointed; anal figs striate; body size: 810–860 × 780–810	*Pergalumna decoratissima* Pérez-Íñigo & Baggio, 1986. Distribution: Neotropical region
–	Rostrum rounded, with lateral tooth on each side; anal figs not striate; body size: 780–962 × 630–747	*Pergalumna paradecoratissima* Ermilov & Kalúz, 2012. Distribution: Ecuador
17	Surface of prodorsum with three striate bands (one basal, transverse and two dorso-lateral, longitudinal striate bands)	18
–	Surface of prodorsum without three striate bands	19
18	Rostrum pointed; posterior part of notogaster with striate bands; body size: 415–464 × 282–332	*Pergalumna boliviana* Ermilov, 2013 (see Ermilov and Niedbala 2013). Distribution: Bolivia
–	Rostrum rounded; posterior part of notogaster without striate bands; body size: 278 × 213	*Pergalumna passimpunctata* Balogh & Mahunka, 1969 (see also Ermilov and Niedbała 2013). Distribution: Brazil
19	Surface of prodorsum with numerous longitudinal stria	20
–	Surface of prodorsum without stria	21
20	Whole surface of prodorsum striate; interlamellar setae minute; notogastral porose areas *Aa* rounded; body size: 630–697 × 448–514	*Pergalumna striatiprodorsum* sp. n. Distribution: Costa Rica
–	Only basal part of prodorsum striate; interlamellar setae of medium size; notogastral porose areas *Aa* elongated transversally; body length: 750	*Pergalumna magnipora magnipora* (Hammer, 1961). Distribution: Peru
21	Interlamellar setae of medium size or long	22
–	Interlamellar setae minute or represented by alveoli	30
22	Rostrum tridentate; body size: 384 × 360	*Pergalumna plumata* Pérez-Íñigo & Baggio, 1986. Distribution: Neotropical region
–	Rostrum rounded	23
23	Notogastral porose areas *A1* of specific structure	24
–	Notogastral porose areas *A1* rounded or oval	25
24	Body surface microgranulate; notogastral porose areas *Aa* weakly triangular, *A1* long, band-shaped, specifically curving; body size: 332–352 × 246–266	*Pergalumna elongatiporosa* sp. n. Distribution: Costa Rica
–	Body surface smooth; notogastral porose areas *Aa* elongate oval, *A1* with rounded anterior part (with distinct margins) and triangular posterior part (without distinct margins); body size: 448–498 × 315–348	*Pergalumna sura* P. Balogh, 1997 (see also Ermilov et al. 2014). Distribution: Neotropical region
25	Interlamellar setae longer than bothridial setae	26
–	Interlamellar setae shorter than bothridial setae	27
26	Adanal setae of medium size, *ad*_3_ inserted posteriorly to lyrifissures *iad*; surface of prodorsum with small tubercles; body size: 1062–1261 × 713–863	*Pergalumna paralongisetosa* Ermilov & Kalúz, 2012. Distribution: Ecuador
–	Adanal setae minute, *ad*_3_ inserted laterally to lyrifissures *iad*; surface of prodorsum without tubercles; body size: 697–713 × 498–506	*Pergalumna ecuadorensis* Ermilov & Kalúz, 2012. Distribution: Ecuador
27	Notogastral porose areas *A1* clearly larger than other porose areas; body length: 400	*Pergalumna melloi* Pérez-Íñigo & Baggio, 1994. Distribution: Brazil
–	Notogastral porose areas *A1* not larger than other porose areas	28
28	Two pairs of notogastral porose areas (*A1*, *A2*) observed, *A1* very narrowly band-shaped; body size: 394–410 × 295	*Pergalumna horvathorum* P. Balogh, 1997. Distribution: Neotropical region
–	Three or four pairs of notogastral porose areas observed, *A1* rounded or oval	29
29	Three pairs of notogastral porose areas (*A3* not observed) present; median pore absent; body size: 390–420 × 342–360	*Pergalumna pauliensis* Pérez-Íñigo & Baggio, 1991. Distribution: Brazil
–	Four pairs of notogastral porose areas (including *A3*) present; median pore present; body size: 410–490 × 315–365	*Pergalumna aequalis* (Sellnick, 1923). Distribution: Neotropical region
30	Notogastral porose areas *Aa* rounded	31
–	Notogastral porose areas *Aa* elongated transversally	32
31	Rostrum pointed; notogastral porose areas *A1* and *A3* oval or rounded; body length: 620	*Pergalumna bifissurata* Hammer, 1972. Distribution: Polynesia and Galapagos Islands
–	Rostrum rounded; notogastral porose areas *A1* and *A3* elongated; body length: 500–540	*Pergalumna australis* Pérez-Íñigo & Baggio, 1980. Distribution: Brazil and Ecuador
32	Four pairs of notogastral porose areas present; interlamellar setae represented by alveoli; body size: 276–348 × 228–300	*Pergalumna parva* Pérez-Íñigo & Baggio, 1986. Distribution: Brazil
–	Three pairs of notogastral porose areas present (*A2* absent); interlamellar setae represented by microsetae	33
33	Lamellar setae longer than rostral setae; surface of anterior part of prodorsum granulate; body size: 863–1000 × 680–697	*Pergalumna silvatica* Hammer, 1961 (see also Ermilov et al. 2014). Distribution: Neotropical region
–	Lamellar setae shorter than rostral setae; surface of prodorsum not granulate; body size: 468 × 408	*Pergalumna aegra* Pérez-Íñigo & Baggio, 1986. Distribution: Brazil

^1^ We did not include two Neotropical species, *Pergalumna obvia* (Berlese, 1914) and *Pergalumna curva ventralis* (Willmann, 1931) (sensu Subías 2004, updated 2014), in the key. We consider *Pergalumna obvia* as representative of the genus *Galumna* Heyden, 1826 without additional studying of the type material (see Ermilov et al. 2013b). *Pergalumna curva* ventralis has no lamellar lines (see Hammer 1958, 1961, 1972), therefore we consider this species as representative of the genus *Allogalumna* Grandjean, 1936 (see generic diagnosis for *Allogalumna* in Ermilov et al. 2013a).

## Supplementary Material

XML Treatment for Pergalumna elongatiporosa

XML Treatment for Pergalumna striatiprodorsum
